# A cross-sectional survey of water and clean faces in trachoma endemic communities in Tanzania

**DOI:** 10.1186/1471-2458-11-495

**Published:** 2011-06-24

**Authors:** Morgan Rog, Bonnielin Swenor, Luis C Cajas-Monson, Wilson Mchiwe, Steven Kiboko, Harran Mkocha, Sheila West

**Affiliations:** 1 Dana Center for Preventive Ophthalmology, Wilmer Eye Institute, Johns Hopkins University, Baltimore, MD. USA; 2 Kongwa Trachoma Project, Kongwa, Tanzania

## Abstract

**Background:**

Face washing is important to interrupt the transmission of trachoma, the leading infectious cause of blindness worldwide. We aimed to assess the household and personal factors that affected water use and face washing practices in Kongwa, Tanzania.

**Methods:**

We conducted a household water use survey in 173 households (329 children) in January, 2010. Self reported data on water use practices, observed water in the household, and observed clean faces in children were collected. Contingency table analyses and logistic regression analyses were used to measure associations between unclean faces and risk factors.

**Results:**

We found that women are recognized as primary decision makers on water use in a household, and respondents who reported laziness as a reason that others do not wash children's faces were significantly more likely to have children with clean faces. Washing was reported as a priority for water use in most households. Sixty four percent (95% Confidence Interval = 59%-70%) of children had clean faces.

**Conclusions:**

Attitudes toward face washing and household water use appear to have changed dramatically from 20 years ago when clean faces were rare and men made decisions on water use in households. The sources of these attitudinal changes are not clear, but are positive changes that will assist the trachoma control program in strengthening its hygiene efforts.

## Background

Trachoma is the leading infectious cause of blindness worldwide [1]. Though this disease is no longer a problem in most developed countries, it remains a significant issue in much of the developing world where it most seriously affects poor, rural communities [1]. The main population of concern for active trachoma in communities is preschool-aged children who are the reservoirs of infection. After repeated infections over a lifetime, they have the potential to become blind from the disease [1].

The Dodoma region of central Tanzania is among the areas in Africa most affected by trachoma [2]. Previous research has shown that there are numerous risk factors for trachoma in this population [3]. Face washing, or clean faces, among preschool-aged children has been shown to be associated with a significant decrease in risk for trachoma [4,5]. While increasing distance from the household to water has been associated with increasing risk of trachoma, the critical association may be the relationship of trachoma with the decreasing amount of water in the house that is allocated to washing [6].

Previous studies had been carried out to determine predictors for washing among children under the age of five years. Studies have indicated that although distance to water was a factor in predicting clean faces, water supply alone did not explain face washing habits among households [7-9]. Rather, there were a number of perceptions and societal impediments that affected ability to clean children. For example, the mother's perception that face washing a single child required at least one liter of water, and was less important than other uses for household water supplies, were shown to be the most important factors in predicting unclean faces among children under five years old [7-9].

These studies also showed that women were unlikely to reprioritize water use on their own because ultimately men were in charge of making household decisions [9]. Women also felt that they would face criticism for washing their children's faces instead of doing their other household tasks, which were often seen as more important. Additionally, there was a widespread consensus that it would not be beneficial to wash one's own children if the other children that they played with were not washed [8]. Women also feared that washing their children's faces would make them appear to be asserting their superiority in the community [9]. These studies were carried out prior to implementation of a trachoma control program, part of whose effort was behavior change communication to enhance face washing of children. After ten years of a National Trachoma Control program, we felt it instructive to return to this population and reassess water use patterns and perceptions about use.

The purpose of this study was to reassess water use patterns in households in Kongwa, more than 20 years after the original studies, to determine household and personal risk factors for an unclean face.

## Methods

### Population

The household water use survey was carried out in six villages in Kongwa in January 2010 (survey attached as Additional File 1). Notably, this year the rains had started earlier than usual. Data were collected from 173 households with children under age five years, and included data on 329 children under the age of five years. All households in a village were divided haphazardly but equally among 6 trained census takers. The nature of this study required extensive probing, based on answers provided by the household respondents. Two of the census takers spoke English as well as the local language, and thus could communicate with the two English-speaking investigators responsible for probing questions, and with the household respondents. For this reason, all households assigned to these two interviewers, and which contained children ages under five years, were part of this study. The houses assigned to the interviewers were not preselected on any criteria except being in the villages evaluated by the census during January.

We aimed for at least 150 households but took every eligible household in the six villages. Our study had 80% power to detect an OR of 0.3 for factors that are associated with clean faces, assuming a design effect of 1.5.

### Questionnaire

The household water use survey that was conducted focused on factors which have been shown in previous studies to be risk factors for trachoma. These include: type of water source and distance from the household to the water source, decision-making regarding usage of household water, and face washing practices. Respondents could be either parent of the child (children) under age five years.

Specifically, the survey queried respondents regarding the type of water source used (well, rain water catch, local lake, bought water) and distance to that water source (< 30 minutes, 30 minutes-1 hour, > 1 hour-1.5 hours, > 1.5 hours-2 hours, > 2 hours) during both the dry and rainy seasons. Open-ended questions were asked pertaining to household water use (drinking, cooking, washing clothes or dishes, washing hands or faces), and general household water use decision making (male head of household, female head of household, or female spouse as decision-maker). Investigators also queried whether children above age five were allowed to use household water for drinking or washing on their own, or whether they had to ask permission. In addition, the survey included open-ended questions asking about face washing practices, ascertaining reasons why village children do not have their faces washed and how often children in the household have their faces washed (< 1/day, 1/day, > 1/day). Finally, investigators posed open-ended questions about how village leaders and village members could ensure that children's faces were washed.

### Procedure

Working in conjunction with project personnel conducting a census update, researchers surveyed households with children under the age of five years. Over a three week period, researchers and project personnel visited houses in order to conduct the household water use survey. There were two teams consisting of an investigator and census team member that visited every household to assess eligibility and gain consent for the survey. Village health workers accompanied the teams, introducing the Tanzanian project personnel and researchers to household members in order to build rapport. After gaining consent, the interviewer started with the census and the survey questions. During the process, researchers also recorded their observations of facial cleanliness of the children under age five. Three signs were taken as an indicator of an unclean face: eye crusting, nasal discharge, and the presence of flies on the child's face, any one of which was sufficient to be classified as an unclean face [10]. In addition to recording answers to survey questions, researchers also recorded observations of household water containers and estimated the amount of water in the house. A typical plastic bucket holds 20 liters so water was estimated in units of 20 liters.

Researchers attempted to reconcile any discrepancies between their observations and responses to survey questions by asking additional probative questions. For example, if respondents stated that water had already been obtained for the day but containers were empty, the interviewers further probed as to other sources where water was kept. Notably, if children's faces were unclean but the respondent stated the face had been washed that day, further questions on why the child was unclean as observed were asked.

### Data Analysis

Answers to open ended survey questions were categorized and coded. Rainy season water source categories included well, rain water catch, local lake, purchased water, and water pump, and dry season water sources included well, rain water catch, local lake, purchased water, water pump in another village, and water pump within the village. Reasons that other children's faces are not washed included: mother is too busy, water is too scarce, perception that washing is not important, laziness of the mother, children do not want to be washed, mother forgets to wash children, and several other reasons that were stated by few persons. Additionally, respondents' reasons for the presence of unclean faces were coded into the following categories: child was sick, mother had to leave early and was not able to wash the child's face, the child's face became unclean after having already been washed, and various other reasons stated by few persons.

Contingency table analysis was used to examine bivariate associations between unclean faces and the survey responses. Logistic regression analyses with generalized estimating equations were used to determine odds ratios (OR) and 95% confidence intervals (CI) for associations between unclean faces and selected variables. For these models, the child was the unit of analysis, as this method allows for the adjustment of the correlation between children residing in the same household [11]. Regression models were optimized using stepwise methods and only contained variables maintaining a p-value of 0.05 or lower when included in the model. Potential confounders considered included: total amount of water stored at the household (L), age of the child (< 1, 2-4, 5), sex of the child, other children in the household, decision making about household water use for washing and drinking among children over 5 years (decide on their own or must ask), and reasons village children's faces are not washed: water is too scarce (y/n), mother is lazy (y/n), washing child is not important to mother (y/n), mother is too busy to wash children (y/n). The final model included the total number of children less than 7 years of age within the household, age of the child (< 1, 2-4, 5), and if the respondent indicated that other mother's laziness was a reason for village children's unclean faces (yes/no). Data were analyzed using SAS (Statistical Analysis System Institute, Cary, NC).

This study received ethical approval from the Johns Hopkins University School of Medicine Institutional Review Board and the National Institute for Medical Research in Tanzania. Written informed consent from each respondent was obtained prior to interview and observations.

## Results

None of the 173 household refused to participate in the survey. Of the 329 children under age 5 years in the households, 60 were not observed during the survey, primarily because they were not at home during the time of the interview. Unobserved children tended to be mostly female (62%) and between the ages of four and five years (47%) (Table 1).

**Table 1:** Characteristics of observed (n = 269) and unobserved (n = 60) children under 5 years old

Characteristic	Observed	Unobserved
**Gender**	**n(%)**	**n(%)**

Male	144 (53.5%)	23 (38.3%)

Female	125 (46.5%)	37 (61.7%)

*X^2^*p value		**0.03**

**Age**	**n(%)**	**n(%)**

< 1 year	63 (23.4%)	11 (18.3%)

1-2 years	81 (30.1%)	6 (10.0%)

2-3 years	48 (17.8%)	8 (13.3%)

3-4 years	55 (20.5%)	7 (11.7%)

4-5 years	22 (8.2%)	28 (46.7%)

*X^2^*p value		**< 0.0001**

More than half of the households with children (58%, 95% Confidence Interval (CI) = 49%-64%) had 6 or more people, and contained 269 children under the age of five years residing within the household (Table 2). The majority of head of households (57%, 95% CI = 47%-64%) did not have formal education, although some (39%) household heads had between six and twelve years of education (Table 2). In the dry season, 70% (95% CI = 63%-77%) of respondents reported being 30 minutes or more to a water source, and 13% reported being more than 2 hours (Table 2).

**Table 2:** Participant household characteristics

Education of Head of Household (years)*	n (%)
None	95 (56.6%)
1-6 years	8 (4.8%)
6-12 years	66 (38.7%)
**Number of Children < 5 years old**	
1	82 (47.4%)
2-3	86 (49.7%)
> 3	5 (2.9%)
**Number of People in Household**	
3-5	75 (43.3%)
6-10	88 (50.9%)
> 10	10 (5.8%)
One-Way Distance to Water (Dry Season) **	
< 30 min	52 (30.0%)
30 min-1 hr	57 (33.0%)
1-1.5 hr	11 (6.4%)
1.5-2 hr	29 (16.8%)
2+ hr	22 (12.7%)
Household Water Source in Rainy Season***	
Rain water	60 (34.7%)
Buy Water	41 (23.7%)
Local Lake	39 (22.5%)
Well	31 (17.9%)
Water pump	2(1.2%)
Household Water Source in Dry Season***	
Buy Water	93 (53.8%)
Well	30 (17.3%)
Water pump	11 (6.4%)
Pump in another village	31 (17.9%)
Other	8 (4.6%)

*4 people were missing education information

**2 people were missing distance to water information

***participants may report more than one source

Main sources of water varied, with 35% of respondents listing rain water, 24% purchasing water, 23% obtaining water from a local lake (which fills in the rainy season), and 18% using a well, as shown in Table 2. In the dry season, the sources shift, with many respondents reporting buying water. The cost of purchased water was between 25 and 30 Tanzanian shillings (approximately $.02) for 20 liters of water.

About 23% of households had less than 20 liters of water in the house, and 24% of households had more than an estimated 100 liters (Table 3). The majority of respondents (66%) listed early morning as the best time to collect water, with the second most frequent time being in the late afternoon. Reported water usage for different activities varied little across households. Possible activities were supplied by the respondent and included drinking, cooking, washing clothes and dishes, and washing hands, face or body. Four respondents did not initially list washing as a typical use for household water, explaining later that they had either forgotten to list it or use another source of water for bathing. Several respondents listed livestock as another use of their household water resources.

**Table 3:** Household level water use survey responses

Water Use Survey Questions	Frequency (%)
Observed Amount of Water stored in House (L)*	
< 20	39 (22.8%)
20-40	47 (27.5%)
40-60	16 (9.4%)
60-80	18 (10.5%)
80-100	10 (5.9%)
100+	41 (24.0%)
Decision maker for Household Water Use Priorities*	
Female (male spouse is head of household)	125 (72.3%)
Female (female is head of household)	45 (26.0%)
Male Head of Household	1 (0.6%)
**Frequency of Face Washing**	
< 1/day	3 (1.7%)
1/day	68 (39.3%)
> 1/day	102 (59.0%)
Reason Other Children's Faces are not Washed**	
Water too scarce	121 (69.9%)
Mother is Lazy	27 (15.6%)
Mother thinks it's not important	95 (54.9%)
Mother too busy	74 (42.8%)
Mother refuses to wash children	6 (14.6%)
Children don't want to be washed	2 (4.9%)
Mother forgets to wash children	2 (4.9%)
No Soap	2 (4.9%)

*2 households missing a response

**persons allowed multiple free responses

All but one of the respondents listed the mother as responsible for making decisions regarding household water use (Table 3). Of these, 145 had a male head of the household and in 45 a female was the head of the household. One respondent listed the father as responsible for decisions regarding household water use. In this particular household, the male respondent indicated that the female could make water use decisions if he was travelling or when the family did not have to pay for water. Most respondents, 86%, reported that children over the age of five years could get water for drinking on their own but 77% of respondents indicated children had to ask to get water for washing.

Of the 269 children observed in this study, 64% (95% CI = 59% to 70%) had a clean face. Most children were unclean due to the presence of dried nasal discharge; relatively few (9%) had flies on the face or ocular crusting (4%). Most respondents (59%, 95%CI = 52%-66%) reported that the faces of children under five years were washed more than once a day (Table 3). According to these respondents, the best times of day to wash children's faces are early in the morning, and early afternoon. Of the 40% of respondents who reported washing their children once per day the majority listed early morning as the best time. Two percent of respondents reported washing the faces of children under the age of five years less than once per day.

When asked about the main reasons that faces of children in the village were not washed, respondents gave a variety of explanations. The primary reasons were water scarcity, parents not understanding the importance of washing, and the mother's laziness, as shown in Table 3. Respondents were also asked what village leaders and villagers could do to ensure that all children's faces are washed. The majority (86%) of respondents suggested that village leaders should take steps to provide more water in the village to encourage face washing. Most respondents (68%) replied that village members could try to educate each other about the importance of face washing in order to increase this practice but some (77%) also raised concerns about telling their neighbors what to do.

A total of 93 children had unclean faces although their parent claimed to have washed their children's faces. When we probed for this incongruity, various reasons were provided (Table 4). Some respondents claimed that they had already washed the child, but he or she had subsequently become unclean with 53% explaining that the child was sick and 23% reporting that the child had become unclean while playing after having been washed. Other respondents reported that although they usually washed their children in the morning they were not able to do so that day, with 21% reporting that the mother had to leave early to go to the farm and thus did not have an opportunity to wash her child's face.

**Table 4:** Stated reasons for observing unclean faces in children, in subset of parents reporting the child's face was washed (n = 93)

Reason	Frequency (%)
Child is Sick	37 (52.86%)
Child got dirty after being washed	16 (22.43%)
Mother had to leave before child woke up so face was not washed	15 (21.43%)
Other	2 (2.86%)

Contingency table analysis was conducted to examine bivariate relationships between survey question responses and the presence of an unclean face (Table 5) The youngest and oldest children were less likely to be unclean. The percentage of unclean faces increased with increasing numbers of other pre-school age children in the house. Those who reported the reason for not washing faces was laziness were more likely to have a clean child. A multivariate logistic regression with unclean faces as the dependent variable was created to determine the independent contribution of these household and personal factors to the presence of unclean faces (Table 6). The number of children within a household under the age of seven years was shown to increase the risk of unclean faces (Odds Ratio = 1.6, 95% CI = 1.2-2.1) (Table 6). Additionally, children ages 2-4 years were at greatest risk of an unclean face; children under 2 years had a decreased risk (Odds Ratio = 0.1, 95% CI = 0.07-0.4) and children older than four years had a decreased risk (Odds Ratio = 0.3, CI = 0.08-0.9) (Table 6). Finally, children were more likely to have clean faces in the households where the respondent listed "laziness of care provider" as a reason that other children in the village had unclean faces (Odds Ratio = 0.3, 95% CI = 0.1-0.7). Sources of water (rainy season p value = 0.33; dry season p value = 0.10) and walking time to water source (rainy season p value = 0.34; dry season p value = 0.10) were not associated with unclean faces. The total amount of water at each household had a null association with unclean faces in univariate analyses and multivariate analyses. Therefore, these variables were not included in the final model (Table 6).

**Table 5:** Univariate associations of household and personal factors with presence of unclean faces in children (n = 269)

Age of Child	% unclean	Odds Ratio	95% CI
< 1 years	14.3%	0.8	0.2-2.7
1-2 years	43.2%	3.4	1.1-11.0
2-3 years	43.8%	3.5	1.03-11.9
3-4 years	49.1%	4.3	1.3-14.5
4-5 years	18.2%	REF	REF
**# other pre-school age children in household**	**% unclean**	**Odds Ratio**	**95% CI**
1	24.4%	REF	REF
2	33.3%	1.5	0.7-3.5
3	37.7%	1.9	0.8-4.4
4+	54.8%	3.8	1.4-10.3
**Amount of water stored in Household (L)**	**% unclean**	**Odds Ratio**	**95% CI**
< 40	28.2%	0.5	0.3-0.99
40-60	66.7%	2.6	1.1-6.8
60-80	32.0%	0.6	0.2-1.7
80-100	8.3%	0.1	0.02-1.00
100+	42.7%	REF	REF
**Reasons Other Children's faces are not washed**	**% unclean**	**Odds Ratio**	**95% CI**
Water too Scarce: yes	34.8%	1.1	0.7-1.9
no		1.0	
Mother is Lazy: yes	17.0%	0.3	0.1-0.7
no		1.0	
Mother thinks it's not important:yes	37.3%	0.9	0.5-1.4
no		1.0	
Mother too busy: yes	37.4%	0.9	0.5-1.5
no:		1.0	

**Table 6:** Multiple regression model of factors associated with an unclean face in children

Parameter	OR (95% CI)
# pre-school age children	1.6 (1.2, 2.1)

child < 2 years old	0.1 (0.07, 0.4)

child 2-4 years old	1.00

child 5 years old	0.3 (0.08, 0.9)

Reason for other children's unclean faces is mother's laziness	0.3 (0.1, 0.7)

## Discussion

The results of the survey indicate that household water was plentiful and the distance to water is relatively short. This is in contrast to the 1986 survey, conducted in the dry season, which indicated that 37% of households had to travel at least two hours to reach their main water source [12]. However, the current household water use survey was conducted during the rainy season, and a significant amount of rainfall had already occurred by the time the survey was administered. This explains the reported use of rainwater collection and local lakes for household water sources, as compared to the dry season, in which buying water or using a pump in another village were more predominant. Thus, although household water was plentiful at the time of the survey, water availability may still be an issue during the dry season. Approximately half of households surveyed reported purchasing water in the dry season, which is a significant change from previous studies, where purchasing water was not an option [13]. There was no means to transport water in sufficient quantities to sell in 1986, whereas oxcarts that haul water are plentiful now and a quarter of respondents purchase water, even in the wet season. According to respondents, water costs between 25 and 30 Tanzanian shillings (approximately $.02) for 20 liters. Assuming a family uses 20 liters (one bucket) a day for a year, this is approximately 2% of income, which is estimated to be approximately $442 per year in Tanzania [14]. Taken together, these findings suggest that water availability has improved over the 20 years in the villages in this district and this is an important prerequisite for any hygiene behavior change program. One of the serious limitations raised by community members in a trial of improving face washing is the limitation of water for use for hygiene purposes (7).

The previous studies also indicated that even if women did want to change the way water use was prioritized within the household they may not be able to due to their husband's control over household decision-making [8]. In contrast, our present study found the majority of households reported that either the female (in households with both males and females) or the female who is the head of household made decisions regarding household water use. Only one household reported the man as the decision-maker. This represents a significant shift in household decision-making as compared to previous years, and may potentially explain why face washing has become more of a priority within households and children's faces are cleaner. Any program to improve face washing behavior for trachoma control needs to determine if mothers have control over water use for hygiene purposes, and if not, must consider addressing the allocation of water from a family (including the male) standpoint.

Face washing appears to have moved up as a household water use priority. Previous studies indicated that the major impediment to face washing was the mother's perception that face washing required a lot of water and was not as important as other household water uses such as cooking and drinking [9]. In this study on the other hand, the vast majority of households spontaneously listed washing as one of the major uses of their water and many households reported washing their children more than once per day. There appeared to be a general consensus that washing the faces of young children was an important priority. These results are in contrast to previous studies where women reported not wanting to wash their children's faces for fear of appearing that they were trying to be superior to their neighbors (9). In fact, many respondents in this study reported thinking that mothers who did not wash their children were lazy. This prioritization of face washing to the point of thinking that others who do not do so are lazy was significantly associated with a reduction in unclean faces, indicating the shift in village norms with respect to attitudes about face washing. The source of this shift in norms is unclear, as there has been spotty national radio programs exhorting the importance of face washing for trachoma control and some material presented in primary schools over the years. Children must be clean to attend schools, and perhaps over the years this practice has affected the new mothers of young children in the present study. However, intensive village level programs were not part of this effort. There has also been strengthening of Maternal and Child Health Programs, resulting in widespread use of health cards with birthdates and vaccinations recorded. These cards are much valued as they allow access to services, and focus attention on well child practices. Other programs in the area include increasing access to HIV/AIDS testing and treatment and increasing small business such as petrol-driven small grain milling. Thus, attributing this shift in norms around face washing and water use to any one cause in our study is not possible. Also, it is uncertain when the attitudinal shift occurred, but the long time frame between this and previous surveys suggest that behavior change may take more than a few years to occur.

The proportion of clean faces in this study also differed from previous studies. A 1989 study which involved extensive household surveys, revealed that only 13% of children observed had clean faces [4]. However in this study, 64% of children observed had clean faces. This proportion is comparable to a recent study which found 62% of children observed in the home to have clean faces [15]. This upward trend in the proportion of children with clean faces suggests that face washing has increased in priority among households. The proportion of unclean faces we observed may have even been higher, as parents reported washing the children's faces but because they were sick the nasal discharge had returned. We continued to count this as unclean face as the point is not to wash the face a certain number of times but to ensure the child has a clean face, even if it takes more washing to accomplish that goal.

The total number of pre-school age children per household, and whether children were between the ages of 2 and 4, were both found to be significantly associated with the presence of unclean faces within a household. This is a logical result, given that the mother is responsible for keeping all of her children's faces clean. Therefore, the more children she has under school age, the less time she has to keep each one of them clean Children in a household were more likely to be clean if the respondent listed "laziness of mother" as a reason for not washing. This is also a reasonable finding as it indicates that in those households face washing is seen as so important that one would be considered "lazy" if they were not to wash their children's faces.

There were several limitations to this study. We did not observe 60 children under the age of five because the children were not at home at that time. These missing children were predominantly female and approximately five years old (Table 1). Since they were not observed, it is impossible to know whether they had clean faces. If they were less clean than those children that were observed, the protective effect of age would have been less pronounced. However, previous studies have also found that older children are more likely to have clean faces, so there is no reason to think that these older children were more likely to have unclean faces than those observed [10,13,15]. Additionally, this study was conducted during the rainy season, which certainly accounted for the increased availability of water among the household surveyed. This increased water availability may, in turn, account for the high proportion of children with clean faces. However, the rainy season results in children crowded together in houses and is the season associated with more upper respiratory illnesses. This may explain why nasal discharge was the preponderant sign as an indicator of an unclean face, and suggests that the proportion of clean faces when not in rainy season may have been even higher. Finally, we did not record the time of day the observations were made, although visits in the village were uniformly made between 9 am and 4 pm. Time of day, as well as rain that day, may impact how much water is stored.

## Conclusions

The results of this study suggest that there have been significant changes in attitudes towards household water use and face washing in the Kongwa district of central Tanzania, which is positive news for trachoma control. Despite the limitations of this study, it is clear that women are now recognized as the primary decision-makers regarding household water resources. Similarly, face washing is now widely recognized as a priority among households, representing a significant shift from previous surveys conducted over the past twenty years. In addition the proportion of unclean faces has dropped, and the finding that unclean faces are much less frequent in households where respondents feel it is a mark of laziness suggests that societal attitudes may help educational messages improve hygiene behavioral change interventions.

## Competing interests

The authors declare that they have no competing interests.

## Authors' contributions

All authors have read and approve the final version of the manuscript.

MR was responsible for designing data collection, overseeing data collection, participating in data analyses and writing the manuscript. BS conducted data analysis, and reviewed drafts of paper. LCM oversaw data collection and reviewed drafts of manuscript. WM and SKa collected data and reviewed the manuscript. HM trained data collection team, helped draft and review manuscript. SWb designed study, secured funds, helped draft and revise manuscript, and wrote 16 pages of replies to reviewers.

## Pre-publication history

The pre-publication history for this paper can be accessed here:

http://www.biomedcentral.com/1471-2458/11/495/prepub

## Supplementary Material

Additional file 1 **Household Water Use Survey PRET**. This is the survey carried out in six villages in Kongwa in January 2010. Click here for file
