# Supplementary material for: A cross-sectional survey of water and clean faces in trachoma endemic communities in Tanzania
Source: BMC Public Health. 2011 Jun 24;11:495. doi: 10.1186/1471-2458-11-495 (PMC3141459; doi:10.1186/1471-2458-11-495)
Supplement: Additional file 1 — Household Water Use Survey PRET. This is the survey carried out in six villages in Kongwa in January 2010. [file 1471-2458-11-495-S1.PDF]

Household Water Use Survey

HID:

Respondent CID:

Date of interview: __ __ / __ __ / __ __ __ __

Time of interview: __ __ : __ __ (24 hour clock)

Respondent Name: ______________________

Number of children less than age 5 in census: _____

Start by observing the faces of the children in the household ages <5 years during census.

A. OBSERVE: *Did the mother start washing children as you came up to house?*

1 ☐ Yes 2 ☐ No

B. OBSERVE: *Number of faces age less than 5 years observed:* _____

C. OBSERVE: *Answer each of the following questions for each face observed:*

**Child's CID:**	**Eye crusting?**	**Nasal Discharge?**	**Flies on face?**
C1.	1 ☐ Yes 2 ☐ No	1 ☐ Yes 2 ☐ No	1 ☐ Yes 2 ☐ No
C2.	1 ☐ Yes 2 ☐ No	1 ☐ Yes 2 ☐ No	1 ☐ Yes 2 ☐ No
C3.	1 ☐ Yes 2 ☐ No	1 ☐ Yes 2 ☐ No	1 ☐ Yes 2 ☐ No
C4.	1 ☐ Yes 2 ☐ No	1 ☐ Yes 2 ☐ No	1 ☐ Yes 2 ☐ No
C5.	1 ☐ Yes 2 ☐ No	1 ☐ Yes 2 ☐ No	1 ☐ Yes 2 ☐ No

Introduction: "We are interested in issues around water and water use in this village, and would like to ask you a few questions about water for your household".

1. May we see where you store your water?

OBSERVE: 1a. *Number of storage containers:* _____

1b. *Estimate size of largest container:* _____ (liters)

1c. *Estimate the total amount of water in the household:* _____ (liters)

2. Where do you go to get your water?

1 ☐ Well
2 ☐ Rain water catch
3 ☐ Local lake
4 ☐ Buy water
5 ☐ Other, specify: ______________________

3. Do you have to pay anything for this water? Either when you get it, or some other fee?

1 ☐ Yes —If Yes,→ 3a. How much do you have to pay? ______________ (shillings)
2 ☐ No

4. How long does it take you to walk one way from this house to this water source?

1 ☐ < 30 minutes
2 ☐ 30 minutes to 1 hour
3 ☐ >1 hour to 1.5 hours
4 ☐ >1.5 hours to 2 hours
5 ☐ More than two hours

5. Do you use another source of water in the dry season?

1 ☐ Yes — If Yes, → 5a. What is that source of water?

2 ☐ No

5a. What is that source of water?

- 1 ☐ Well
- 2 ☐ Rain water catch
- 3 ☐ Local lake
- 4 ☐ Buy water
- 5 ☐ Other, specify: ____________

5b. How long does it take you to walk one way from this house to this water source?

- 1 ☐ < 30 minutes
- 2 ☐ 30 minutes to 1 hour
- 3 ☐ >1 hour to 1.5 hours
- 4 ☐ >1.5 hours to 2 hours
- 5 ☐ More than two hours

6. Have you or a member of the household already gone for water today?

1 ☐ Yes

If yes, OBSERVE:

- 1 ☐ *Containers are mostly full, SKIP TO QUESTION #7*
- 2 ☐ *Containers are mostly empty, ASK the following:*

6a. Have they already come back with water for today?

- 1 ☐ Yes
- 2 ☐ No, they have not come back yet

2 ☐ No

If no, ASK... 6b. At what time of day do you or someone in the house usually go to get water?

- 1 ☐ Early morning (still dark to dawn)
- 2 ☐ Late morning (dawn to noon)
- 3 ☐ Early afternoon (noon to 3 pm)
- 4 ☐ Late afternoon (3pm to dusk)
- 5 ☐ Evening (dusk to dark)

7. RECONCILE AMOUNT OF WATER IN THE HOUSE

If they have already just gotten water, but none is there, ASK:

7. I see that you have already gotten water, yet none/very little is in the container.
Can you tell me what you used the water for already?

Else, ASK the following:

7. What do you use this water for? →

	Prompt after each answer, "anything else?" until they say "nothing else". Mark all unused answes as NO.		
a.	Drinking?	1 ☐ Yes	2 ☐ No
b.	Cooking?	1 ☐ Yes	2 ☐ No
c.	Washing clothes or dishes/objects	1 ☐ Yes	2 ☐ No
d.	**Washing hands or faces (body)**	1 ☐ Yes	2 ☐ No
e.	Other, specify:		

8. Water is pretty scarce in this area, and we know families use it wisely.
Who makes most of the decisions in this house about for what activities water can be used, like washing?

1 ☐ Head of household (male)

8a. Respondent? 1 ☐ Yes 2 ☐ No

PROBE: for when the women can make decisions...

8b. What if the (name of head of household) is away traveling, can (name of female) make decisions about water use?

1 ☐ Yes 2 ☐ No

8c. Are there other situations where the woman can make decisions about water use? Please explain: ______________________

2 ☐ Head of household (female)

8a. Respondent? 1 ☐ Yes 2 ☐ No

3 ☐ Spouse (female)

8a. Respondent? 1 ☐ Yes 2 ☐ No

4 ☐ Other, specify: ______________________

9. Can children above age 5 years, for example, decide if they want water for drinking, or must they ask an adult in this household?

1 ☐ They can decide
2 ☐ They must ask
3 ☐ Other, specify: ______________________

10. Can children above age 5 years, for example, decide if they want water for washing, or must they ask an adult in this household?

1 ☐ They can decide
2 ☐ They must ask
3 ☐ Other, specify: ______________________

Interviewer: If question #7 mentions washing hands or face, then SKIP to question #12.
If question #7 does NOT mention washing hands or face, then ASK question #11.

11. You did not mention that you use the water I saw for washing hands or faces of children.
Is there another source of water that your household uses for washing hands and faces?

1 ☐ Yes

If yes, ASK: 11a. Where is that source of water; may I see it?

1 ☐ *Source is another container in or around the house*
2 ☐ *Source is outside the house-a lake, or well, where you have to walk*
3 ☐ *They Refuse or Do Not Know*

2 ☐ No

If no, ASK: 11b. RECONCILE: Is it something that is done infrequently? Please explain:

____

12. We understand that washing children is another chore, and uses water, which may be a problem.
What are the main reasons you feel that children do not have their faces or hands washed every day?

	Prompt after each answer, "anything else?" until they say "nothing else". Mark all unused answers as NO.	
a.	Too busy with other chores	1 ☐ Yes 2 ☐ No
b.	Water too scarce to use for washing	1 ☐ Yes 2 ☐ No
c.	No need to wash children - don't see importance	1 ☐ Yes 2 ☐ No
d.	Danger to children in washing every day	1 ☐ Yes 2 ☐ No
e.	Other, specify: *RECONCILE: Make sure it makes sense as a reason.*	

13. How often does (name of child that you observe) have his/her face washed with water in a day?

1 ☐ Less than 1/day
2 ☐ 1 per day → 13a
3 ☐ More than 1/day → 13b

13a. When is the best time of day to wash the face?

1 ☐ Early morning (still dark to dawn)
2 ☐ Late morning (dawn to noon)
3 ☐ Early afternoon (noon to 3 pm)
4 ☐ Late afternoon (3pm to dusk)
5 ☐ Evening (dusk to dark)

13b. When are the best times of day to wash the face?

	Mark all unused answers as NO:	
a.	Early morning (still dark to dawn)	1 ☐ Yes 2 ☐ No
b.	Late morning (dawn to noon)	1 ☐ Yes 2 ☐ No
c.	Early afternoon (noon to 3 pm)	1 ☐ Yes 2 ☐ No
d.	Late afternoon (3pm to dusk)	1 ☐ Yes 2 ☐ No
e.	Evening (dusk to dark)	1 ☐ Yes 2 ☐ No

14. RECONCILE: *If kid has dirty eyes or nose, and allegedly has already been washed by time of interview, then PROBE:* Is there a reason why (Name of kid) does not seem clean now?

____

15. What could the leaders of this village do to make it easier for mothers of children to wash their children's faces each day? What would you like to see happen to make it easier?

____

____

RECONCILE ANSWER: *Make it clear that we are asking about what leadership could do.*

16. Are there things that the people of the village could do to make it easier to keep young children clean in this village?

____

____

RECONCILE ANSWER: *Make it clear that we are asking about what the people or families themselves could do.*

Finish: "Thank you for your time and effort."
